# Case Report: Pediatric group A streptococcal toxic shock syndrome complicated by acute necrotizing tracheobronchitis requiring veno-venous extracorporeal membrane oxygenation

**DOI:** 10.3389/fped.2026.1900220

**Published:** 2026-10-21

**Authors:** Hongsheng Deng, Kun Chen, Ke Chen

**Affiliations:** Department of Critical Care Medicine, Jinhua Municipal Central Hospital, Jinhua, Zhejiang, China

**Keywords:** extracorporeal membrane oxygenation, metagenomic next-generation sequencing, necrotizing tracheobronchitis, pediatric intensive care, point-of-care ultrasound, streptococcal toxic shock syndrome, *Streptococcus pyogenes*

## Abstract

Group A streptococcal toxic shock syndrome (STSS) in children is uncommon and can progress rapidly to multi-organ failure. Acute necrotizing tracheobronchitis (NTB) is a rare airway complication of severe infection and has seldom been reported during extracorporeal support. We describe a previously healthy 7-year-old girl admitted with 6 days of fever and 3 h of chest tightness and dyspnea. She developed septic shock, severe pneumonia, acute respiratory distress syndrome (ARDS), disseminated intravascular coagulation (DIC), acute kidney injury, and multi-organ dysfunction. Blood and sputum cultures were negative at 48 h. Metagenomic next-generation sequencing (mNGS) of blood and bronchoalveolar lavage fluid identified *Streptococcus pyogenes*. Serial fiberoptic bronchoscopy was used for airway clearance and extubation assessment. Procedure records described progression from mucosal congestion to erosive necrosis with pseudomembrane-like plaques, followed by healing included broad-spectrum antimicrobials, corticosteroids, intravenous immunoglobulin (IVIG), tocilizumab, hemoperfusion, continuous renal replacement therapy (CRRT), and veno-venous extracorporeal membrane oxygenation (ECMO) from hospital days 2 to 9. Lung ultrasound during ECMO showed confluent B-lines with regional A-lines. Ultrasound images were acquired on day 3; a day-4 progress note recorded a left ventricular ejection fraction (LVEF) of 62% and mild tricuspid regurgitation. In the setting of markedly elevated troponin, these findings favored sepsis-associated myocardial injury over fulminant myocarditis. ECMO was discontinued on day 9, CRRT on day 10, and the patient was extubated on day 13 after bronchoscopy showed improved airway necrosis. She was discharged on hospital day 28, 27 days after admission. At follow-up, an intratracheal granulation-tissue lesion was excised endoscopically, and the family reported preserved neurocognitive function. Together, these findings show that NTB can complicate pediatric group A streptococcal STSS and that serial bronchoscopy may help guide ventilator and ECMO weaning; as one case without a comparison group, it does not establish which treatments were responsible for recovery.

## Introduction

Group A *Streptococcus* (*Streptococcus pyogenes*) causes disease ranging from pharyngitis to necrotizing fasciitis. STSS is defined by isolation of the organism from a normally sterile site together with hypotension and multi-organ failure (1), and mortality remains substantial (2). This child presented during the 2022–2023 upsurge in pediatric invasive GAS disease, which renewed attention to severe complications even though measured severity did not clearly rise alongside incidence (3).

Severe group A streptococcal pneumonia can progress to ARDS requiring ECMO (4). NTB has most often occurred with influenza/*Staphylococcus aureus* co-infection or *Mycoplasma pneumoniae*, and is not well described in group A streptococcal STSS (5; 6; 7). It is likely under-recognized because diagnosis requires bronchoscopy, which is not routine early in pediatric septic shock. Unrecognized pseudomembrane sloughing can cause sudden, life-threatening obstruction (5; 7). A recent pediatric STSS case in this journal reported ARDS after adenotonsillectomy without airway necrosis or bronchoscopic findings (8).

We report a 7-year-old girl with group A streptococcal STSS, extreme hypercytokinemia, and multi-organ dysfunction syndrome (MODS), managed with veno-venous ECMO, CRRT, hemoperfusion, immunomodulatory therapy, and serial fiberoptic bronchoscopy, which identified acute NTB. The case adds to the limited literature on NTB in pediatric STSS and describes how bronchoscopic findings informed the timing of support withdrawal.

## Case description

A previously healthy 7-year-old girl was admitted to the pediatric intensive care unit (PICU) at Jinhua Maternal and Child Health Care Hospital on 19 October 2023 (Table 1). She had a 6-day history of intermittent fever, worsening over the preceding 3 days, and 3 h of chest tightness and dyspnea, with no chronic illness or immunodeficiency. ECMO and continuous renal replacement therapy (CRRT) were provided by teams from Jinhua Municipal Central Hospital.

**Table 1:** Clinical timeline during index hospitalization (19 October–15 November 2023).

Hospital day	Date (2023)	Key events
1	Oct 19	Admission; intubation; empiric linezolid, vancomycin, and meropenem; septic shock resuscitation
2	Oct 20	Bronchoscopy; mNGS sampling; V-V ECMO (04:38); CRRT; hemoperfusion; methylprednisolone started
3	Oct 21	mNGS result: *S. pyogenes*; methylprednisolone escalated; second hemoperfusion; lung and cardiac ultrasound images acquired
4	Oct 22	Peak IL-6 942,990 pg/mL; progress note records LVEF 62% and mild TR; bronchoscopy
7	Oct 25	Bronchoscopy: erosive necrosis with pseudomembranes
9	Oct 27	ECMO decannulation; bronchoscopy
10	Oct 28	CRRT discontinued
12	Oct 30	Bronchoscopy: necrosis and old bleeding improved
13	Oct 31	Extubation; bronchoscopy: focal erosion above the carina only
16	Nov 3	Transfer out of PICU; chest CT: pulmonary inflammation partially resolved; tracheal lumen patent on 3D reconstruction
19	Nov 6	Ward: supplemental oxygen discontinued
28	Nov 15	Discharge (27 days after admission)

Hospital day 1 is the day of admission. Tocilizumab 240 mg was prescribed during the acute phase; the administration date is not documented and is therefore not placed on this timeline (Supplementary Table S2).

CRRT, continuous renal replacement therapy; ECMO, extracorporeal membrane oxygenation; IL-6, interleukin-6; LVEF, left ventricular ejection fraction; mNGS, metagenomic next-generation sequencing; TR, tricuspid regurgitation; V-V, veno-venous.

On admission she weighed 19 kg, with temperature 36.3 °C, heart rate 167 beats/min, respiratory rate 50 breaths/min, and blood pressure 82/55 mmHg; oxygen saturation remained 90%–93% on 8 L/min by mask. She was lethargic and in marked respiratory distress, with suprasternal and intercostal retractions, cyanotic lips, and diffuse crackles. Extremities were cool with capillary refill over 5 s, and non-blanching petechiae, purpura, and confluent ecchymoses covered the trunk and limbs. Severe pneumonia with septic shock and respiratory failure was the working diagnosis; skin findings and coagulation studies indicated disseminated intravascular coagulation (DIC), with an International Society on Thrombosis and Haemostasis overt-DIC score of 7 (9).

She was intubated on admission and ventilated in assist-control mode; fluid resuscitation and norepinephrine were started per pediatric sepsis guidelines (10). Invasive arterial pressures ranged from 46 to 87/28–63 mmHg, and copious frothy secretions were suctioned from the endotracheal tube. Delivered tidal volumes were 40–50 mL (2.1–2.6 mL/kg) with high resistance on manual ventilation, reflecting severe pulmonary edema and poor lung compliance. No portal of entry was identified, and no antecedent trauma, varicella, or skin infection was recorded.

Arterial blood gas obtained before ECMO cannulation (FiO₂ 1.0) showed pH 7.11, PaCO₂ 53.2 mmHg, PaO₂ 27.8 mmHg, lactate 7.4 mmol/L, and base excess −12.4 mmol/L. At a mean airway pressure of 35 cmH₂O, the oxygenation index was 125.9—well above pediatric ECMO thresholds—prompting veno-venous ECMO cannulation later that day.

Laboratory studies on 20 October revealed white blood cell count 0.52 × 10⁹/L, absolute neutrophil count 0.20 × 10⁹/L, platelet count 26 × 10⁹/L, international normalized ratio 3.55, activated partial thromboplastin time 150.1 s, fibrinogen 1.96 g/L, D-dimer 28,870 µg/L, troponin I > 50 ng/mL, creatinine 167.5 µmol/L, and aspartate aminotransferase 638 U/L (serial trends in Supplementary Tables S1–S1b). Fluid balance over the first 24 h was 4030 mL in against 490 mL out, with urine output of 0.48 mL/kg/h, meeting criteria for oliguric acute kidney injury (AKI).

## Diagnostic assessment

### Microbiological assessment

mNGS (Illumina NextSeq shotgun sequencing, MatriDx assay, ∼24-hour turnaround; full methodology in Supplementary Methods) of peripheral blood and BALF, sampled on hospital day 2, detected *S. pyogenes* in both specimens. *M. pneumoniae* was also detected in BALF (significance discussed below). Gram staining of a respiratory specimen showed abundant neutrophils and Gram-positive cocci in pairs and short chains (Figure 2A; Supplementary Figure S2). Blood and sputum cultures were negative at 48 h, and blood cultures remained negative at 5 days. Whether the first set preceded the initial antibiotic dose is not documented, so antibiotic pretreatment cannot be excluded as a cause. A respiratory virus panel and SARS-CoV-2 testing were negative. Results are collated in Supplementary Table S5.

### Imaging assessment

Serial chest radiographs demonstrated bilateral pulmonary opacities and pleural effusions that gradually improved. On lung ultrasound (LUS) acquired on day 3, some zones had confluent B-lines and others A-lines, indicating uneven aeration (11) (Supplementary Figure S4; Figure 1A). The same session confirmed the ECMO cannula within the vessel lumen (Figure 1B) and included cardiac views (Supplementary Figure S3). The LVEF of 62% and mild tricuspid regurgitation (TR) were documented in the progress note dated 22 October (day 4), not measured from the submitted still images (Supplementary Table S7). Preserved systolic function made fulminant myocarditis unlikely despite the high troponin; we therefore used the term sepsis-associated myocardial injury.

**Figure 1:**
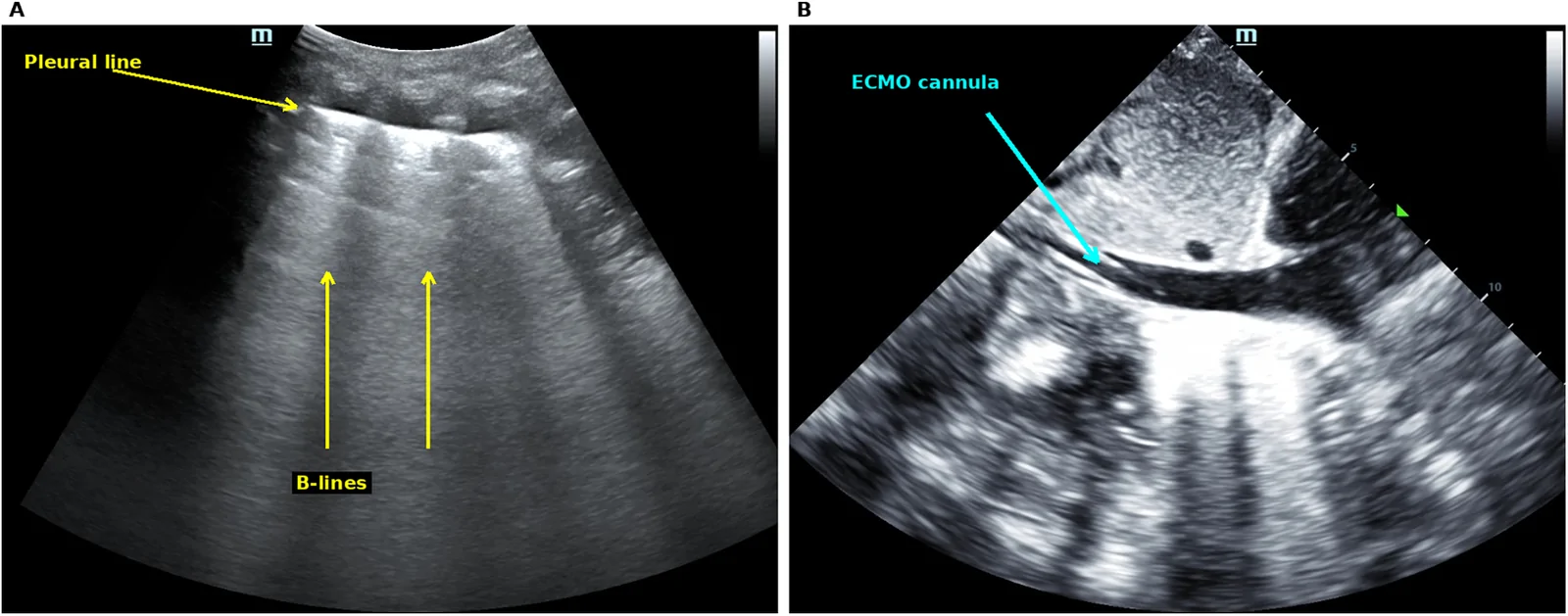
Point-of-care ultrasound on hospital day 3 during veno-venous ECMO. **(A)** Lung ultrasound (convex probe) showing confluent B-lines (arrows) arising from the pleural line, indicating an interstitial pattern; A-lines were present in other zones (full panel in Supplementary Figure S4). **(B)** Bedside ultrasound confirming ECMO cannula position (labeled hyperechoic double-line structure within the vessel lumen). Cardiac ultrasound images from the same session are shown in Supplementary Figure S3; the LVEF and tricuspid regurgitation values are taken from the day-4 progress note, not measured from those images.

### Bronchoscopic airway assessment

Serial fiberoptic bronchoscopy via the endotracheal tube is summarized in Supplementary Table S3. On day 2 the carina was sharp and the bronchi congested and edematous with viscous secretions; by day 4 the mucosa was hyperemic with bleeding and purulent secretions. By day 7, during ECMO support, erosive necrosis with yellow, moss-like pseudomembranes and blood staining extended throughout the tracheobronchial tree, and by day 9 both tracheal and bronchial mucosa had marked erosive necrosis with old hemorrhage. At the day 13 (31 October) examination the carina remained sharp, both bronchi were patent, and the mucosa was pale with focal erosion above the carina but no petechiae or mass lesions. Chest CT on day 16 (3 November) found partial resolution of bilateral pulmonary inflammation and no tracheal narrowing on 3D reconstruction. Endoscopic still images were not retained; the findings above come from procedure notes.

### Diagnostic reasoning

Fever, purpura, shock, and multi-organ injury prompted consideration of meningococcemia and other causes of toxic shock, but cultures identified no meningococcus and mNGS detected *S. pyogenes* in blood and BALF. MIS-C was less likely: there was no evidence of recent SARS-CoV-2 infection, and respiratory failure dominated over gastrointestinal or coronary involvement (12). The conjunctival, oral, and lymph-node findings expected in Kawasaki disease-shock syndrome were absent. Cytopenias and raised ferritin also prompted consideration of HLH, but the ferritin value was not retained in the available record, a complete HScore and the HLH-2004 criteria could not be applied, and no genetic testing was performed (13).

## Therapeutic interventions

### Antimicrobial therapy

Linezolid, vancomycin (0.38 g, approximately 20 mg/kg every 8 h), and meropenem (0.38 g, approximately 20 mg/kg every 8 h) were started empirically on admission, before the cause of septic shock was known. Vancomycin was reduced on 20 October to 0.25–0.285 g (approximately 13–15 mg/kg) every 6–8 h, but troughs on 23 and 26 October were 46.9 and 32.9 µg/mL, both well above the 10–20 µg/mL target, in a child receiving CRRT for AKI. mNGS identified *S. pyogenes* on 21 October, and the three-drug regimen continued unchanged rather than moving to a penicillin- and clindamycin-based regimen (14); the chart gives no reason for this. Meropenem continued through the acute phase and linezolid after transfer to the ward; neither drug's stop date is documented (Supplementary Table S2).

### Extracorporeal and renal support

Veno-venous ECMO was established at 04:38 on 20 October for refractory hypoxemia. An 18-French drainage cannula was placed in the right femoral vein (30 cm) and a 17-French return cannula in the right internal jugular vein (10 cm), with a Maquet PLS-i oxygenator (Supplementary Figure S1). Blood flow and sweep gas flow were both approximately 2 L/min, the blood flow corresponding to about 105 mL/kg/min. Heparin was titrated to an activated clotting time of 160–180 s (circuit details in Supplementary Table S4).

After cannulation, lung-rest settings on day 2 were an inspiratory pressure of 12 cmH₂O above a PEEP of 12 cmH₂O, FiO₂ 0.60, and a rate of 20/min; oxygen saturation was 86%–94%. Later ventilator settings were not charted at the same level of detail. CRRT ran at a blood flow of 100 mL/min (approximately 5 mL/kg/min) for oliguric AKI and fluid overload, and was stopped on day 10 as urine output and renal function improved. Systemic coagulation results during CRRT are in Supplementary Table S1b; the effluent dose and the circuit's own anticoagulation protocol were not charted.

### Immunomodulatory and adjunctive therapy

Methylprednisolone was started on day 2 at 38 mg (approximately 2 mg/kg) every 12 h. During the period of marked hyperinflammation it was increased on day 3 to 190 mg (approximately 10 mg/kg per dose) every 12 h, a pulse-range 20 mg/kg/day. It was reduced to 38 mg once daily from day 6 as shock and inflammatory markers improved. IVIG was prescribed at 10 g per dose (approximately 0.53 g/kg) and tocilizumab as Actemra 80 mg/4 mL, 12 mL (240 mg; approximately 12.6 mg/kg per dose), matching the 12 mg/kg weight-based dose used for cytokine release syndrome in children under 30 kg (15). For neither agent are the number of doses or administration dates recorded, so cumulative exposure cannot be calculated and we report prescribed doses rather than daily regimens. The stated indications were cytokine-storm control for methylprednisolone, presumed superantigen neutralization for IVIG, and IL-6 receptor blockade as rescue therapy for tocilizumab. Of these, only corticosteroids and IVIG have guideline standing in STSS, and IL-6 blockade could blunt a beneficial inflammatory response. Ulinastatin and sivelestat were given for protease and neutrophil-elastase inhibition, and blood products, albumin, mannitol, milrinone, and norepinephrine as supportive care; vasoactive doses, and the indications for milrinone and mannitol, are not recorded. Hemoperfusion with an HA330-II resin cartridge (Jafron Biomedical, Zhuhai, China) was performed twice, on 20 and 21 October, for cytokine and toxin adsorption. Peak procalcitonin was >100 ng/mL and IL-6 942,990 pg/mL.

## Follow-up and outcomes

ECMO was stopped and the cannulas were removed on hospital day 9 after review by vascular surgery. CRRT ended the following day. By day 13, radiographs and airway findings had improved enough for extubation. High-flow nasal cannula (HFNC) oxygen at 15 L/min and FiO₂ 0.30 maintained oxygen saturation above 95%. No immediate complication of decannulation or extubation was documented.

On transfer to the general ward on day 16, temperature was 37.5 °C, heart rate 111/min, blood pressure 119/84 mmHg, and oxygen saturation 99% on nasal oxygen at 3 L/min. PICU diagnoses included STSS, severe streptococcal pneumonia, ARDS, DIC, AKI, sepsis-associated myocardial injury (recorded in the chart as “toxic myocarditis”), MODS, and acute NTB (Supplementary Table S6). Oxygen was discontinued on day 19. She was discharged on 15 November 2023, 27 days after admission (hospital day 28), alert, afebrile, and breathing comfortably, with an occasional cough.

Adverse and unanticipated events were supratherapeutic vancomycin troughs during CRRT, *Candida albicans* in urine and stool treated with fluconazole after prolonged broad-spectrum antibiotics and high-dose corticosteroids, and weakness improving from MRC grade 3 to grade 4 before discharge. A healing wound over the right lower leg, the side used for femoral ECMO drainage, was noted at discharge; the record does not state its cause. Tracheal granulation and scar narrowing became apparent after discharge.

About three months after discharge, she was readmitted from 26 February to 1 March 2024 after persistent throat phlegm and discovery of an intratracheal lesion on imaging. CT pulmonary angiography excluded pulmonary embolism but identified an enhancing lesion near the carina. Bronchoscopy on 28 February found mild scar-like narrowing in the mid-trachea and a polypoid lesion in the lower trachea. The lesion was removed with an electrosurgical snare without complication. Histology demonstrated granulation tissue with hemorrhage and hemosiderin, without malignancy (Figure 2B), indicating post-inflammatory repair rather than recurrent disease. Such lesions usually respond to endoscopic treatment but can recur, so we advise continued endoscopic and pulmonary surveillance.

**Figure 2:**
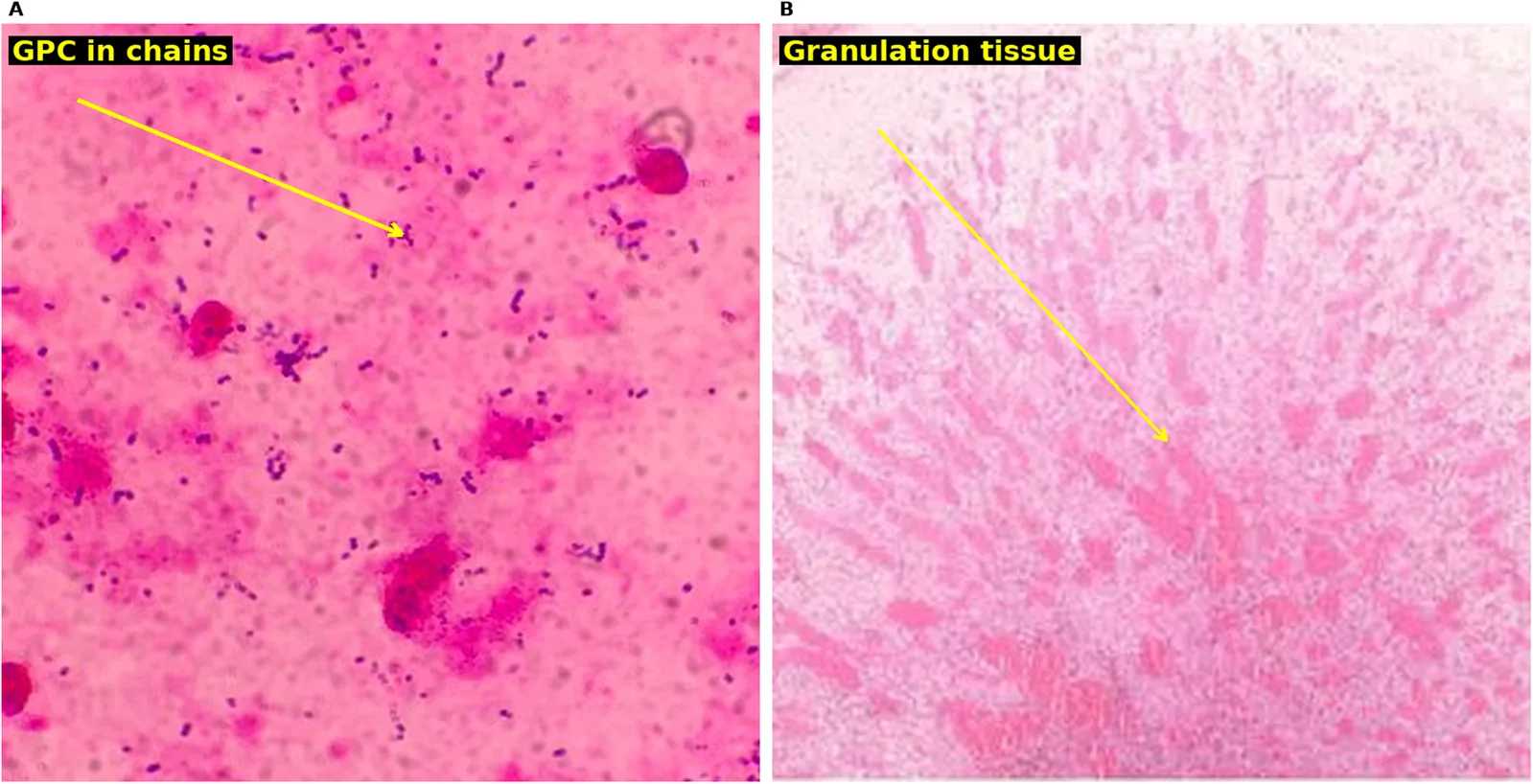
Microbiology and histopathology. **(A)** Gram stain of a respiratory specimen showing Gram-positive cocci in pairs and short chains with neutrophils (also Supplementary Figure S2). **(B)** H&E micrograph of the February 2024 tracheal biopsy showing granulation tissue with hemorrhage and hemosiderin deposition.

She was admitted again from 23 March to 4 April 2024 with bronchopneumonia and a *Klebsiella pneumoniae* urinary tract infection, which resolved with azithromycin followed by piperacillin–tazobactam. At the most recent contact, her guardians reported normal peer interaction, accurate counting and arithmetic, and no need for home oxygen. No standardized neurocognitive assessment, surveillance bronchoscopy, or formal spirometry had been completed.

## Discussion

The case met the clinical criteria for STSS—hypotension with renal, hematologic, hepatic, respiratory, and cutaneous involvement—with two qualifications: the rash was petechial and purpuric rather than the generalized macular erythema of the classic description, and the organism was detected by sequencing rather than isolated (1). Alternatives were set aside as described above (12). The illness fell within the 2022–2023 rise in pediatric invasive GAS disease (3).

### Necrotizing tracheobronchitis and bronchoscopy

In a PICU series of children with influenza A and *S. aureus* co-infection, NTB was recognized only at bronchoscopy (7); endoscopy also permits removal of cast-like debris, and delay risks fatal obstruction from sloughed membrane (5). Dense necrosis may require repeat bronchoscopy alongside antibiotics and corticosteroids (16).

Here, serial bronchoscopy tracked progression from congested mucosa to erosive necrosis with pseudomembranes and then improvement before ECMO removal and extubation. Unlike the earlier pediatric STSS case in this journal (8), here the airway lesion itself governed the timing of weaning.

Why the airway necrotized cannot be established from this case, but three mechanisms are plausible and probably additive: direct mucosal injury from the streptococcal exotoxins and superantigens that drive the systemic syndrome; mucosal ischemia during prolonged vasopressor-dependent shock with DIC; and mechanical injury from intubation and the high airway pressures needed before cannulation. Without acute-phase histology, none of these can be confirmed in this patient.

### Extracorporeal support, renal replacement, and cardiac findings

Veno-venous ECMO was started for hypoxemia refractory to conventional ventilation, as used in pediatric severe ARDS (4), and confluent B-lines on LUS during support reflected alveolar–interstitial involvement. An LVEF of 62% with only mild TR does not match the profound ventricular failure that usually drives arterial cannulation, so veno-arterial support was not chosen. On the same grounds the chart label of “toxic myocarditis” is better read as sepsis-associated myocardial injury.

### Antimicrobial strategy

IDSA guidance for severe invasive GAS disease pairs a *β*-lactam with a protein-synthesis inhibitor to suppress toxin production (14), and observational data associate clindamycin—with or without IVIG—with lower mortality (17; 2). The regimen used here met that structure in principle, since linezolid is a protein-synthesis inhibitor given alongside meropenem, but it was not the guideline-preferred penicillin plus clindamycin combination, and vancomycin added a second anti-staphylococcal agent with no activity advantage against *S. pyogenes*. The chart does not record why the regimen was left unchanged once the pathogen was known.

### Immunomodulatory adjuncts

Of the agents added for hypercytokinemia, only IVIG has observational support in invasive GAS disease (2); the case for tocilizumab rests on CAR T-cell cytokine release syndrome rather than on sepsis or STSS data (15). These adjuncts were rescue measures, not standard of care.

### Diagnostic reliance on mNGS and *M. pneumoniae* co-detection

Pathogen identification depended on mNGS. Clinical assays can be sensitive and specific, but performance depends on assay and specimen, and contamination-driven false positives are recognized (18; 19). Because the consensus definition requires isolation from a sterile site (1), a sequencing-only detection places this case closer to a probable than a culture-confirmed diagnosis. We treat *S. pyogenes* as the primary pathogen: purpura, refractory shock, DIC, and its detection in blood—where *M. pneumoniae* was absent—match streptococcal toxic shock. *M. pneumoniae* infection is usually more indolent, no macrolide was given, and recovery occurred without one; but because it has been linked to NTB elsewhere (6), a contributory role remains possible.

### Limitations

This is a single case, and care was divided between two hospitals, so the record is fragmented; most of the missing dosing and duration data follow from that split. Outcome cannot be ascribed to any one intervention. Diagnosis was culture-negative and rested on mNGS without quantitative read counts, and the contribution of *M. pneumoniae* is unresolved. Acute-phase airway histology was not obtained, and no bronchoscopic images were saved; the 2024 biopsy showed granulation tissue only. The regimen was not narrowed to clindamycin after pathogen identification. Follow-up neurocognition was parental report only, without spirometry. Chart gaps include tocilizumab and IVIG schedules, antibiotic stop dates, vasoactive doses, and CRRT effluent dose and circuit anticoagulation.

### Take-home lessons

In a child with GAS-STSS, persistent airway obstruction, bloody secretions, or failed ventilator weaning should prompt consideration of NTB and bronchoscopic inspection of the airway. When cultures are negative and deterioration is rapid, mNGS can return a pathogen within about 24 h, but the result should be reported as sequencing-based rather than as culture confirmation.

## Patient perspective

The parents described the illness as frightening in its speed: within days their daughter went from fever to life support. ECMO, bronchoscopy, and blood purification were distressing, but the team's explanations helped them stay engaged, and extubation on day 13 felt like a turning point. At follow-up they were relieved that she could count, do arithmetic, recognize family, and play normally with peers. The February 2024 readmission was worrisome, but the benign pathology and uneventful recovery were reassuring.

## Data Availability

The original contributions presented in the study are included in the article/Supplementary Material, further inquiries can be directed to the corresponding author.
