# Supplementary material for: Case Report: Pediatric group A streptococcal toxic shock syndrome complicated by acute necrotizing tracheobronchitis requiring veno-venous extracorporeal membrane oxygenation
Source: Front Pediatr. 2026 Oct 21;14:1900220. doi: 10.3389/fped.2026.1900220 (PMC13731197; doi:10.3389/fped.2026.1900220)
Supplement: Supplementary file 1 [file Datasheet1.pdf]

Supplementary Materials: Pediatric Group A Streptococcal Toxic Shock Syndrome Complicated by Acute Necrotizing Tracheobronchitis Requiring Veno-Venous ECMO

Frontiers in Pediatrics — Case Report

Supplementary Figures S1–S4 and Tables S1–S7. Ultrasound identifiers removed. No patient photographs.

Supplementary Figure S1. Maquet PLS-i oxygenator during V-V ECMO

A

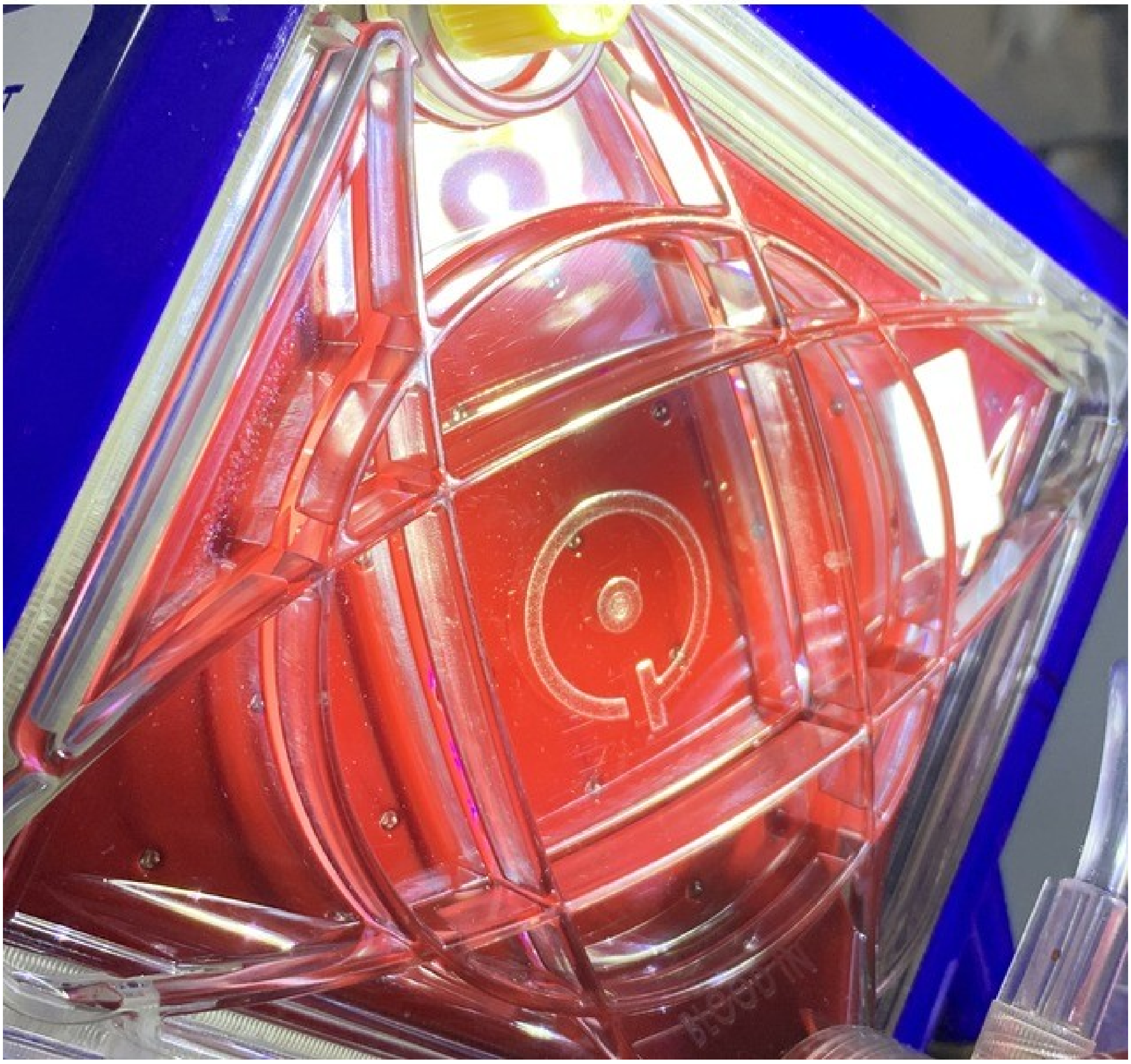

Supplementary Figure S1. Maquet PLS-i membrane oxygenator during veno-venous ECMO (equipment photograph only). Cannulation: right femoral vein 18F (30 cm) drainage; right internal jugular vein 17F (10 cm) return.

Supplementary Figure S2. Gram stain of respiratory specimen

A

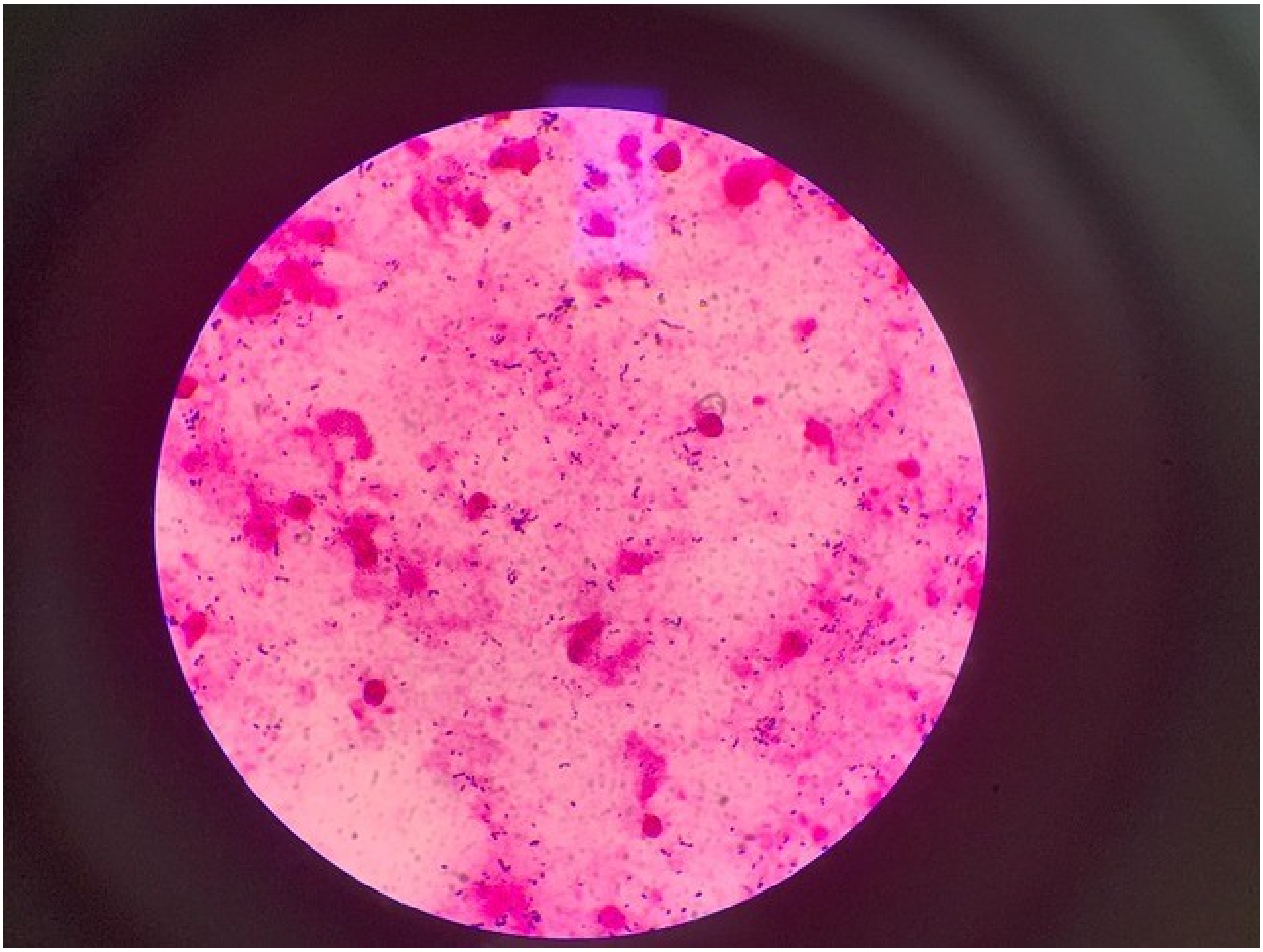

Supplementary Figure S2. Gram stain of respiratory specimen showing Gram-positive cocci in pairs and short chains with neutrophils. Species identification as Streptococcus pyogenes was by mNGS of blood and BALF.

Supplementary Figure S3. Bedside echocardiography during V-V ECMO

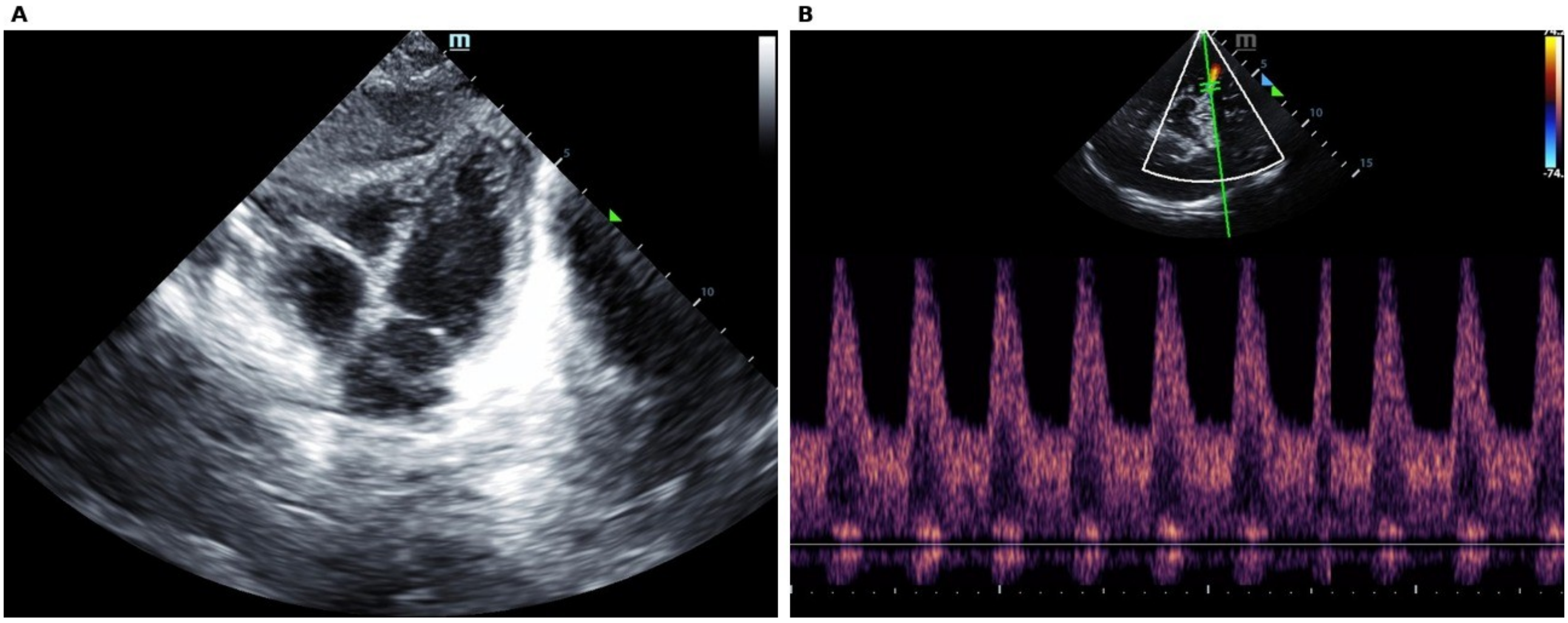

Supplementary Figure S3. Bedside cardiac ultrasound images during veno-venous ECMO (hospital day 3, 21 October 2023). (A) Apical four-chamber view. (B) Spectral Doppler recording from the same image-acquisition session. A progress note dated 22 October recorded LVEF 62% and mild tricuspid regurgitation; these values were not remeasured from the submitted stills. Machine identifiers were removed.

Supplementary Figure S4. Point-of-care lung ultrasound during V-V ECMO

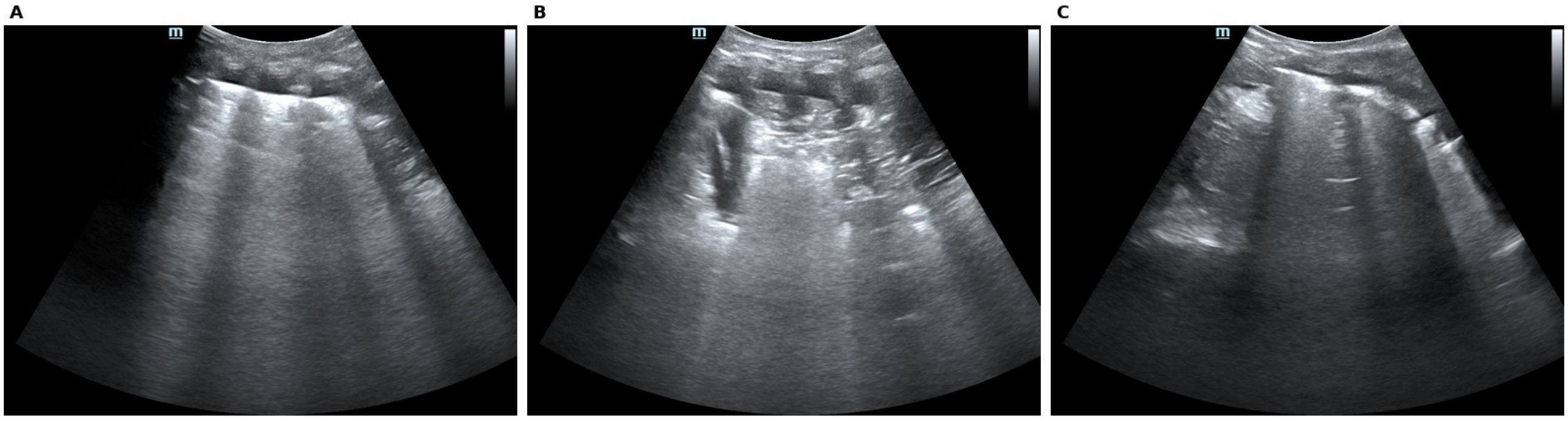

Supplementary Figure S4. Point-of-care lung ultrasound during veno-venous ECMO (hospital day 3, 21 October 2023). (A) Confluent B-lines. (B) Non-confluent B-lines. (C) A-lines. Machine identifiers were removed.

Supplementary Tables and Methods

Supplementary Table S1. Serial key laboratory values

Approximate pediatric reference ranges are shown for orientation.

Date	WBC (×10⁹/L)	ANC (×10⁹/L)	Neutrophils (%)	PLT (×10⁹/L)	Lactate	hs-CRP	PCT	D-dimer	Other
Reference	5.0–13.5	1.5–8.0	Lab-dependent	150–450	0.5–2.2 mmol/L	<5–10 mg/L	<0.05 ng/mL	<500 µg/L	—
19 Oct	2.03	—	—	183	—	—	—	—	—
20 Oct, pre-ECMO blood gas (~04:38)	—	—	—	—	7.4	—	—	—	Oxygenation index 125.9
20 Oct AM	0.52	0.20–0.21	—	26	9.7	65–142	—	28,870	Troponin I >50 ng/mL
20 Oct PM	2.45	—	72.2	57	4.3–5.3	108.91	—	28,870	Troponin I 1.421 ng/mL
22 Oct	19.77	—	88.6	31	1.7	111.56	>100	31,830	IL-6 942,990 pg/mL
23 Oct	—	—	—	—	—	—	—	—	CK 23,200 U/L
06 Nov	7.39	—	—	406	—	33.26	0.20	—	—
10 Nov	11.86	—	49.9	357	—	6.98	—	—	—

Absolute neutrophil count and neutrophil percentage are reported in separate columns and are not interchangeable. Lactate from arterial blood gas analysis before ECMO (7.4 mmol/L) and from the laboratory panel drawn later the same morning (9.7 mmol/L) are separate measurements. Troponin I is reported throughout in ng/mL (1 ng/mL = 1 µg/L). WBC, white blood cell count; ANC, absolute neutrophil count; PLT, platelet count; hs-CRP, high-sensitivity C-reactive protein; PCT, procalcitonin; IL-6, interleukin-6; CK, creatine kinase.

Supplementary Table S1b. Serial coagulation profile

Date	PT (s)	INR	PT activity (%)	Fibrinogen (g/L)	APTT (s)	TT (s)	D-dimer (µg/L)
Reference	10–14	0.8–1.5	70–130	2.38–4.98	24–39	14–21	0–500
20 Oct, 00:51	44.5	3.55	14.70	1.96	150.1	33.1	28,870
21 Oct	15.6	1.24	65.40	3.84	53.5	18.5	24,280
22 Oct	12.5	1.00	100.40	2.67	43.7	18.7	31,830
23 Oct	13.0	1.03	93.10	2.15	46.1	18.9	22,100

The 20 October panel was collected at 00:51, before ECMO cannulation at 04:38; no coagulation panel from 19 October is in the retrievable record. PT, prothrombin time; INR, international normalized ratio; APTT, activated partial thromboplastin time; TT, thrombin time.

Supplementary Table S2. Antimicrobial and immunomodulatory timeline

Body weight 19 kg.

Period	Agents	Notes
From 19 Oct; stop date unavailable	Linezolid	Empiric; continued as consolidation after ward transfer
From 19 Oct; stop date unavailable	Vancomycin 0.38 g (~20 mg/kg) IV q8h	Reduced to 0.25–0.285 g from 20 Oct; troughs 23 and 26 Oct
From 19 Oct; stop date unavailable	Meropenem 0.38 g (~20 mg/kg) IV q8h	Empiric; continued during acute phase
21 Oct	mNGS: *S. pyogenes*	Triple regimen continued; not switched to penicillin/clindamycin
20 Oct	Methylprednisolone 38 mg (~2 mg/kg) IV q12h	
21–23 Oct	Methylprednisolone 190 mg (~10 mg/kg/dose) IV q12h	Escalated
24 Oct onward	Methylprednisolone 38 mg IV once daily	Taper
Date and number of doses unavailable	Tocilizumab order: 240 mg (12 mL; ~12.6 mg/kg) per administration	Actemra 80 mg/4 mL; daily course not confirmed
20–28 Oct	CRRT	Blood flow 100 mL/min
20–27 Oct	Veno-venous ECMO	See Table S4
20–21 Oct	Hemoperfusion	Two sessions
Acute PICU phase; dates/doses unavailable	IVIG 10 g (~0.53 g/kg) per recorded administration	Cumulative dose cannot be calculated
23 and 26 Oct	Vancomycin troughs	46.9 and 32.9 µg/mL; both above the 10–20 µg/mL target
Post-ICU	Linezolid (oral), fluconazole	Fluconazole for *Candida*

Supplementary Table S3. Fiberoptic bronchoscopy summary

Findings from procedure notes. Endoscopic still images were not retained.

Date	Time	Setting	Principal findings	Intervention
20 Oct 2023	02:15	PICU	Congestion/edema; viscous secretions	Suction; respiratory specimen obtained for mNGS
22 Oct 2023	—	PICU	Hyperemia, bleeding, purulent secretions	Documented
25 Oct 2023	15:12	PICU, ECMO	Necrosis, pseudomembrane, blood; BAL	Lavage and suction
27 Oct 2023	08:20	PICU, ECMO	Pale mucosa, erosive necrosis, old bleeding	Monitored
30 Oct 2023	08:36	PICU	Improved necrosis and bleeding	Pre-extubation
31 Oct 2023	—	Pre-extubation	Partial erosion above carina; no active sloughing	Extubation

Supplementary Table S4. ECMO and organ-support details

Item	Detail
Mode	Veno-venous
Cannulation	20 October 2023, 04:38
Drainage cannula	Right femoral vein, 18F, 30 cm
Return cannula	Right internal jugular vein, 17F, 10 cm
Indication	Refractory hypoxemic respiratory failure
Oxygenator	Maquet PLS-i
Anticoagulation	Heparin; ACT 160–180 s
Blood flow / sweep gas	~2 L/min each (1:1); blood flow ~105 mL/kg/min
ECMO end	27 October 2023
Mechanical ventilation	19–31 October 2023
Day-2 lung-rest ventilator settings	Inspiratory pressure 12 cmH_2O above PEEP 12 cmH_2O; FiO_2 60%; RR 20/min; VT 40–50 mL; SpO_2 86–94%
Pre-ECMO oxygenation index	125.9 [(FiO_2 1.0 × MAP 35 × 100) / PaO_2 27.8]
CRRT	20–28 October 2023; blood flow 100 mL/min (~5 mL/kg/min); effluent dose not recorded
Hemoperfusion	20 October (2 h 15 min); 21 October; HA330-II resin cartridge (Jafron Biomedical, Zhuhai, China)

Supplementary Table S5. Metagenomic and microbiological results

Specimen	Method	Result
Peripheral blood	mNGS	*Streptococcus pyogenes*
BALF	mNGS	*S. pyogenes*; *M. pneumoniae* also detected
Respiratory specimen	Gram stain	GPC in chains; neutrophils (Figure S2)
Respiratory virus / SARS-CoV-2	PCR	Negative
Blood culture	Culture	No growth at 48 h and 5 days; timing relative to the first antibiotic dose not documented
Sputum culture	Culture	No growth at 48 h
Stool / urine	Culture	*Candida albicans*

mNGS collected 20 October 2023; reported 21 October 2023 (~24 h). Methods in Supplementary Methods.

Supplementary Table S6. Diagnoses at PICU transfer (3 November 2023) and hospital discharge (15 November 2023)

1. Streptococcal toxic shock syndrome
2. Severe streptococcal pneumonia
3. Acute respiratory distress syndrome
4. Acute respiratory failure
5. Acute kidney injury
6. Disseminated intravascular coagulation
7. Decompensated metabolic acidosis
8. Sepsis-associated myocardial injury (chart label: toxic myocarditis)
9. Electrolyte and metabolic disturbance
10. Hyperlactatemia
11. Leukopenia and neutropenia
12. Secondary thrombocytopenia
13. Hypoalbuminemia
14. Hepatic dysfunction
15. Multi-organ dysfunction syndrome
16. Anemia
17. Acute necrotizing tracheobronchitis
18. Pleural effusion
19. Intestinal flora imbalance

Supplementary Table S7. Bedside ultrasound and echocardiography summary

Date	Day	Modality	Key findings	Therapy
19 Oct 2023	1	Bedside US	No obvious structural cardiac abnormality	Intubation; shock resuscitation
21 Oct 10:38	3	Cardiac US image acquisition	Apical 4-chamber (S3A); no EF/TR assigned to still	V-V ECMO
21 Oct 10:43	3	Lung US	Confluent B-lines; A-lines (S4)	V-V ECMO
22 Oct 2023	4	Cardiac findings in progress note	LVEF 62%; mild TR	ECMO ongoing

Supplementary Methods

mNGS. Peripheral blood and BALF were tested by MatriDx (Jieyi Biotechnology Co., Ltd., Hangzhou, China). BALF (1.2 mL) underwent bead-beating (12,000 × g, 10 min). Blood was centrifuged (1,600 × g, 10 min) for plasma. Nucleic acid was extracted on an automated platform (Xi'an Tianlong; MatriDx Cat# MD049T). BALF nucleic acid underwent PCR-based enrichment (Cat# MD061T); blood DNA proceeded directly to library construction. Libraries were built PCR-free (Cat# MD001T), purified (Cat# MD012), quantified (Cat# MD057), and sequenced on Illumina NextSeq (single-end 50-bp). Human reads (GRCh38.p13) were filtered; remaining reads were aligned to NCBI GenBank and an internal microbial database. Each run included negative and positive controls. A positive call required QC thresholds (library >1 pM, Q20 >85%, Q30 >80%) and either absence in the concurrent negative control or RPM(sample)/RPM(NC) ≥5. Specimens collected 20 October 2023 were reported 21 October 2023. Patient-level read counts were not retained in the clinical report.

Vancomycin monitoring. Troughs on 23 and 26 October 2023 were 46.9 and 32.9 µg/mL.

ECMO. Institutional protocol; heparin anticoagulation with ACT target 160–180 s; blood flow ~2 L/min; sweep gas ~2 L/min (1:1).

Bronchoscopy. Fiberoptic bronchoscopy via the endotracheal tube under sedation. No endoscopic still images were retained.

Point-of-care ultrasound. Lung ultrasound findings classified by B-line and A-line patterns (Volpicelli et al., 2012).
