# Supplementary material for: Case Report: Pediatric group A streptococcal toxic shock syndrome complicated by acute necrotizing tracheobronchitis requiring veno-venous extracorporeal membrane oxygenation
Source: Front Pediatr. 2026 Oct 21;14:1900220. doi: 10.3389/fped.2026.1900220 (PMC13731197; doi:10.3389/fped.2026.1900220)
Supplement: Supplementary file 2 [file Datasheet2.pdf]

CARE Checklist (2016)

CARE Checklist (2016)

Manuscript title: Case Report: Pediatric Group A Streptococcal Toxic Shock Syndrome Complicated by Acute Necrotizing Tracheobronchitis Requiring Veno-Venous Extracorporeal Membrane Oxygenation

Authors: Hongsheng Deng, Kun Chen, Ke Chen
Affiliation: Department of Critical Care Medicine, Jinhua Municipal Central Hospital, Jinhua 321000, Zhejiang, China
Corresponding author: Hongsheng Deng (hs_deng1986@163.com)

Item	CARE checklist item	Reported in revised manuscript
1a	The words “case report” appear in the title	p. 1, Title
1b	The area of focus is described in the title	p. 1, Title
2	Key words	p. 2, Keywords
3a	Abstract — What is unique about this case?	pp. 1–2, Abstract
3b	Abstract — Main symptoms and clinical findings	p. 1, Abstract
3c	Abstract — Main diagnoses, interventions, and outcomes	pp. 1–2, Abstract
3d	Abstract — Main conclusion	p. 2, Abstract
4	Introduction — Background and relevant literature	p. 2, Introduction
5a	De-identified demographic information	p. 3, Case description
5b	Main symptoms and concerns	p. 3, Case description
5c	Medical, family, and psychosocial history	p. 3, Case description (available history: previously healthy; no chronic disease or immunodeficiency; family/psychosocial history not recorded)
5d	Relevant past interventions and outcomes	Not applicable; no pre-admission intervention was documented
6	Clinical findings	pp. 3–4, Case description

Item	CARE checklist item	Reported in revised manuscript
7	Timeline	p. 4, Table 1
8a	Diagnostic methods	p. 5, Diagnostic assessment
8b	Diagnostic challenges	pp. 5–6, Diagnostic assessment
8c	Diagnostic reasoning including differential diagnosis	pp. 5–6, Diagnostic assessment
8d	Prognostic characteristics (where applicable)	p. 6, Diagnostic assessment
9a	Types of intervention	p. 6, Therapeutic interventions
9b	Administration of intervention (dose, strength, duration)	pp. 6–7, Therapeutic interventions; Tables S2 and S4. Unavailable durations are identified explicitly
9c	Changes in intervention (with rationale)	pp. 6–7, Therapeutic interventions and Follow-up and outcomes
10a	Clinician-assessed outcomes	p. 7, Follow-up and outcomes
10b	Important follow-up diagnostic and other test results	p. 7, Follow-up and outcomes
10c	Intervention adherence and tolerability	Inpatient administration described on pp. 6–7; no adherence problem documented. No complication of ECMO decannulation, extubation, or endoscopic excision was recorded
10d	Adverse and unanticipated events	p. 7, Follow-up and outcomes (post-inflammatory tracheal stenosis/granulation tissue and subsequent readmissions)
11a	Strengths and limitations	pp. 7–9, Discussion
11b	Discussion of relevant medical literature	pp. 7–9, Discussion
11c	The rationale for conclusions	pp. 7–9, Discussion
11d	The primary "take-away" lessons	p. 9, Discussion
12	Patient perspective	p. 9, Patient perspective
13	Informed consent	pp. 3 and 9, Case description and Ethics statement

Ethics approval: GA2026-17
Informed consent: Written consent for publication obtained from the patient's legal guardians.
